# Reward Motivation Accelerates the Onset of Neural Novelty Signals in Humans to 85 Milliseconds

**DOI:** 10.1016/j.cub.2009.06.021

**Published:** 2009-08-11

**Authors:** Nico Bunzeck, Christian F. Doeller, Lluis Fuentemilla, Raymond J. Dolan, Emrah Duzel

**Affiliations:** 1 UCL Institute of Cognitive Neuroscience, University College London, 17 Queen Square, London WC1N 3AR, UK; 2 UCL Institute of Neurology, University College London, 17 Queen Square, London WC1N 3BG, UK; 3 Wellcome Trust Centre for Neuroimaging, University College London, 12 Queen Square, London WC1N 3BG, UK; 4 Institute of Cognitive Neurology, Otto-von-Guericke University Magdeburg, Leipziger Strasse 44, 39120 Magdeburg, Germany; 5 German Centre for Neurodegenerative Diseases, Otto-von-Guericke University Magdeburg, Leipziger Strasse 44, 39120 Magdeburg, Germany

**Keywords:** SYSBIO

## Abstract

The neural responses that distinguish novel from familiar items in recognition memory tasks are remarkably fast in both humans and nonhuman primates. In humans, the earliest onsets of neural novelty effects emerge at about ∼150–200 ms after stimulus onset [1–5]. However, in recognition memory studies with nonhuman primates, novelty effects can arise at as early as 70–80 ms [6, 7]. Here, we address the possibility that this large species difference in onset latencies is caused experimentally by the necessity of using reward reinforcement to motivate the detection of novel or familiar items in nonhuman primates but not in humans. Via magnetoencephalography in humans, we show in two experiments that the onset of neural novelty signals is accelerated from ∼200 ms to ∼85 ms if correct recognition memory for either novel or familiar items is rewarded. Importantly, this acceleration is independent of whether the detection of the novel or the familiar scenes is rewarded. Furthermore, this early novelty effect contributed to memory retrieval because neural reward responses, which were contingent upon novelty detection, followed ∼100 ms later. Thus, under the contextual influence of reward motivation, behaviorally relevant novelty signals emerge much faster than previously held possible in humans.

## Results and Discussion

The onset latencies associated with neural novelty responses in recognition memory studies differ largely between nonhuman primates (∼70–80 ms) and humans (∼150–200 ms). Although this large species difference in the speed with which neural novelty signals emerge could potentially be explained by brain size, there is also an important experimental factor that so far has not been fully considered. Nonhuman primates are usually motivated to discriminate novel and familiar stimuli by rewarding them for either detecting the novel item or detecting the familiar item, and in some studies, responses to both novel and familiar items are rewarded [8]. In human recognition memory studies, on the other hand, reward is not used to motivate the detection of novel or familiar items. Remarkably, the possibility that the timing of neural novelty signals might be affected if the discrimination of novel and familiar items is rewarded has not yet been tested. Indeed, novelty processing engages neurotransmitter systems that play an important role in the regulation of motivational aspects of behavior, most notably dopaminergic circuitry [9–12]. Furthermore, reward motivation can energize behavior [13], leading to decreased response times [14] and increased response vigor [15, 16].

In two recognition memory experiments, we used magnetoencephalography (MEG) in humans to test the hypothesis that early novelty responses can be accelerated by reward. Critically, as in nonhuman primate studies of recognition memory, in experiment I the correct detection of either novel or familiar images was rewarded with £0.50, whereas in experiment II subjects discriminated novel from familiar images in the absence of reward.

**Figure 1:**
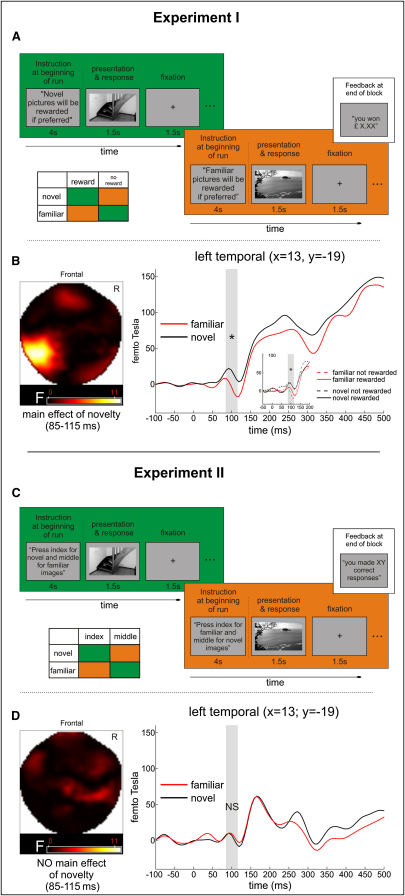
Experimental Design and Results (A and C) Experimental design. Orange and green in the design scheme represent trials in the same run. (B and D) Statistical parametric maps of F-statistics and event-related magnetic fields (ERFs). A difference between ERFs for novel and familiar images emerged over left temporal sensors for the time window 85–115 ms if detecting either the novel or familiar stimulus was rewarded (B, experiment I), but there was no such effect when novelty discrimination was not linked with reward (D, experiment II). Inset in (B) shows all four conditions; there was no interaction between novelty and reward. ^∗^p < 0.005; NS, not significant (p > 0.7).

In experiment I, experimental blocks in which novel images signaled monetary reward (CS+) and familiar images signaled no reward (CS−) alternated with blocks in which familiar images served as CS+ and novel images as CS−. Subjects were informed about the contingency before the beginning of each block and indicated via a button press with their right-hand index or middle finger whether they “prefer” or “do not prefer” the presented image based on the known contingency (Figure 1A). Only correct “I prefer” responses following a CS+ led to a win of £0.50, whereas (incorrect) “I prefer” responses following CS− led to a loss of £0.10. Both correct “I do not prefer” responses following a CS− and (incorrect) “I do not prefer” responses following a CS+ led to neither win nor loss. Importantly, as in nonhuman primate studies, making correct preferences was only possible after correctly discriminating novel and familiar stimuli [17].

In experiment II, subjects indicated the novelty status of images also via a button press with either the index or middle finger of their right hand and the same response apparatus as in experiment I (Figure 1C). In order to match the alternation of response contingencies across blocks in both experiments, the contingency between response finger and novelty status changed from block to block and was also announced at the beginning of each block (Experimental Procedures; see also Figure S1 available online). Furthermore, in both experiments, response-related feedback was given not on a trial-by-trial basis but after the end of an experimental block, and subjects were instructed to respond as accurately as possible as soon as they could classify the stimuli. (See Experimental Procedures for further details about both experiments.)

Behaviorally, subjects' memory performance was equally accurate in both experiments (discriminability index d′ > 2.1) without a significant response bias (β not different from 1) (Table 1). However, responses to novel as well as familiar images were faster in experiment I than in experiment II (p < 0.05 by two-sample t test; see Table 1 legend). MEG data were averaged to event-related magnetic fields (ERFs) and statistically analyzed with SPM8 (Wellcome Trust Centre for Neuroimaging). The time course of ERF differences between conditions was assessed using a priori time windows of interest: 85–115 ms, 115–150 ms, 150–200 ms, 200–500 ms, and 500–700 ms. The first time window (85–115 ms) was motivated by aforementioned animal findings [6, 7]; 150–200 ms, 200–500 ms, and 500–700 ms were chosen based on novelty effects reported in humans [2–5]. In priming studies, but not in recognition memory studies, immediate stimulus repetition responses have also been reported in an early time window from ∼100 to 150 ms [18, 19]. Although these early priming effects were reliable at repetition intervals of less than 100 ms and were not novelty responses (because in these studies, each item was prefamiliarized), for completeness we also considered the time window of 115–150 ms. This was also done to fully characterize the temporal evolution of novelty effects.

**Figure 2:**
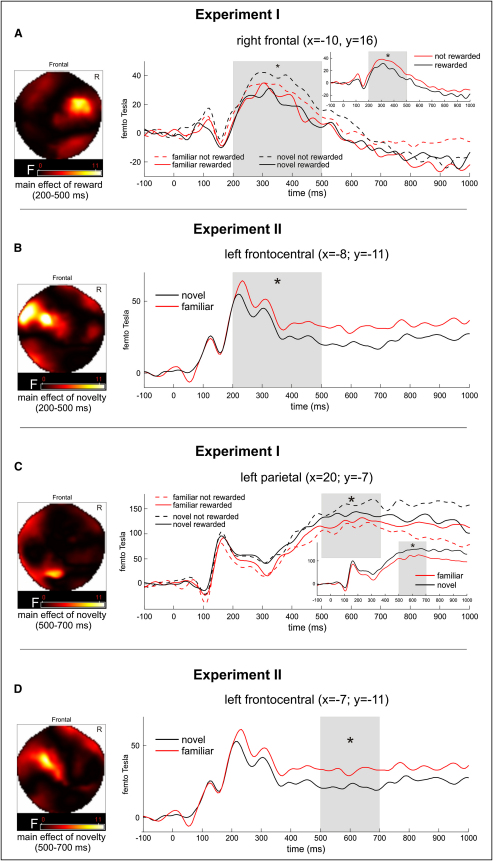
Statistical Parametric Maps of F-Statistics and ERFs (A and C) Experiment I revealed a main effect of reward over right frontal sensors 200–500 ms after stimulus onset (A) and a later main effect of novelty over parietal/occipital sensors 500–700 ms after stimulus onset (C). (B and D) In the absence of reward (experiment II), novelty effects were observed over frontal regions 200–500 ms (B) and 500–700 ms (D) after stimulus onset. ^∗^p ≤ 0.005.

Averaged ERFs for each condition per subject and time window were entered into a second-level random-effects analysis (i.e., experiment I, 2 × 2 analysis of variance [ANOVA] with the factors novelty [novel, familiar] and reward [rewarding, not rewarding]; experiment II, one-way ANOVA with the factor novelty [novel, familiar]). The earliest time window (85–115 ms) revealed a main effect of novelty over left temporal sensors for experiment I in the absence of any interaction (Figure 1B), but no effects for experiment II (Figure 1D). Instead, we observed a “classical” pattern in experiment II of a main effect of novelty for the time window 200–500 ms over frontal sensors (Figure 2B). The same time window (200–500 ms) in experiment I revealed a main effect of reward over right frontal sensors (Figure 2A); this is consistent with the fact that in experiment I, the preference judgment followed an initial old/new discrimination. For the late effect (500–700 ms), both studies revealed a main effect of novelty over either parietal (experiment I; Figure 2C) or frontocentral sensors (experiment II; Figure 2D). Furthermore, for the time window 115–150 ms, experiment I revealed a main effect of novelty over left temporal sensors (Figure S2), but there was no such effect for experiment II. We did observe a main effect of novelty for experiment II for the time window 150–200 ms at a liberal threshold (p = 0.01, uncorrected; F = 9.07; Figure S3), but there were no effects for this time window in experiment I. For a complete list of all effects, see Table S1.

In a subsequent step, we directly compared the novelty responses in both experiments from two sensor locations (temporal and frontal) and time windows (85–115 and 200–500 ms) via 2 × 2 ANOVAs with the factors novelty (novel, familiar; averaged over reward status in experiment I and response finger in experiment II) and the between-subject factor experiment (experiment I, experiment II). In the early time window (85–115 ms; ERFs extracted from the peak over temporal sensors) (Figure 1B), a significant interaction between novelty and experiment (F(1,26) = 5.29, p = 0.03; as well as a main effect of novelty, F(1,26) = 4.39, p < 0.05) with significant differences between ERFs for novel and familiar images in experiment I (p < 0.01 by t test) but not experiment II (p > 0.85 by t test; Figures 1B and 1D) showed a dissociation between early novelty effects and experiments. At the later time window (200–500 ms), there was a main effect of novelty over frontal sensors (F(1,26) = 7.53, p < 0.05) but no significant interaction between novelty and experiment (p > 0.05), suggesting a tendency toward novelty effects in both experiments. The direct comparison of the novelty effects (difference ERFs) between the early time window in experiment I (85–115 ms) and the later time window in experiment II via two-sample t test in SPM did not show any significant differences (p = 0.005 with either unscaled or scaled values). This suggests no statistically significant topography differences between the earliest time window of both experiments. (See Supplemental Data for analyses regarding the topography of old/new effects within experiments.)

Our findings show that in recognition memory tasks, complex novel and familiar stimuli can be neuronally discriminated in humans as early as 85 ms after stimulus onset if detecting either the novel or the familiar stimulus is rewarded. This is ∼70 ms earlier than the earliest novelty effects reported in previous human recognition memory studies, which were in the range of ∼150 ms [2–5]. Importantly, the reward status of stimuli was also already signaled from 200 ms onward. This is remarkably rapid, because in order to signal whether a stimulus predicted reward or not in our paradigm, it was first necessary to determine whether a stimulus was novel or familiar. In fact, the ∼100 ms time difference between the onset of the early novelty response and the reward response is compatible with the possibility that reward status was determined on the basis of the early novelty signal. Therefore, our data strongly suggest that even these very early novelty signals can contribute to the rapid retrieval of behaviorally relevant contingencies. Apart from this novelty-reward contingency, it remains to be established how these novelty signals contribute to conscious or unconscious [20] forms of recognition memory decisions.

Figure 1B and Figure 2B suggest that the earliest ERF novelty responses in both experiments had different topographies (temporal in experiment I, frontal in experiment II). These responses also had different polarities (novel more positive versus familiar more positive), an interesting parallel to findings in nonhuman primates showing that monkey prefrontal novel-familiar response differences have a latency of ∼200 ms and are reversed in polarity compared to early (∼80 ms) temporal responses [8]. However, statistical comparisons of experiments I and II show that topographic differences (∼85 ms versus ∼200 ms; across all contingencies in experiment I as well as experiment II) were not significant. Hence, it remains unclear whether reward motivation merely increased the speed of neural novelty processing or whether it had differential facilitatory effects on the generators of the temporal and frontal novelty responses.

Remarkably, the early novelty effect occurred despite delayed reward feedback and independently of whether novel or familiar items were rewarded (no interaction between novelty and reward), suggesting that facilitation in the context of reward cannot be eliminated by experimentally counterbalancing the contingencies between novelty and reward. It is also remarkable that the effect occurred in the absence of differences in discriminability (d′) and response bias (β).

Behaviorally, subjects responded faster to novel and familiar items in experiment I as compared to experiment II (Table 1). This finding fits well with recent observations of enhanced energization of action through reward as expressed in faster reaction times [14] and increased response vigor [13, 15, 16]. In contrast, discriminability (d′) between novel and familiar items did not differ between experiments. Hence, our data show that reward motivation at retrieval accelerates access to memory representations but does not substantially change the quality of representations accessed. Reward-related improvements in memory accuracy are more likely to be seen for an explicit reward manipulation at encoding (see for example [14, 21]) rather than retrieval.

Taken together, our results show that reward motivation accelerates neural novelty processing and provide a framework for understanding differences between human and nonhuman primate studies of recognition memory. More generally, our findings indicate the importance of studying effects of motivation on the chronometry and functional anatomy of cognitive processes. Although the precise physiological mechanisms for reward-motivated facilitation of very early novelty processing remain to be established, one possibility is that elevated levels of dopamine in the context of reward may play a role [16]. Given the very early onset of novelty responses, context-driven tonic effects of dopamine, for example related to behavioral or attentive set [22, 23], are likely to provide a more plausible mechanism than stimulus-driven phasic effects of dopamine [16, 24].

## Experimental Procedures

### Subjects

Fourteen adults participated in experiment I (eight females and six males; age range 19–31 years, mean age 22.5 years, SD 4.26 years); another group of fourteen adults participated in experiment II (eleven females and three males; age range 19–29 years, mean age 22.71 years, SD 3.97 years). All subjects were healthy and right handed and had normal or corrected-to-normal visual acuity. None of the participants reported a history of neurological, psychiatric, or medical disorders or any current medical problems. Subjects gave written informed consent according to the approval of the local ethics committee (University College London).

### Experimental Design and Task

In both experiments, three sets of (1) a familiarization phase followed by (2) a MEG scanning session (containing both contingencies in random order) were performed. New images were used for each set, resulting in 60 novel and 60 familiar images being used altogether.

(1) Familiarization: in both experiments, subjects were initially familiarized with a set of 20 indoor and 20 outdoor images. Here, each picture was presented twice in random order for 1.5 s with an interstimulus interval (ISI) of 3 s, and subjects indicated via a button press the indoor/outdoor status with their index or middle finger of the right hand.

(2) MEG scanning session: subsequently, a 9 min MEG scanning session of either a recognition memory-based preference judgment task (experiment I) or a recognition memory task in the absence of reward (experiment II) was performed. In both cases, the MEG scanning session was further subdivided into two blocks, each containing 20 images from the familiarization phase (referred to as “familiar images”) and 20 images not previously presented (referred to as “novel images”) (subjects could pause for 20 s between blocks).

In experiment I, novel or familiar images served as either positive (CS+) or negative (CS−) reinforcers. Thus, in any given block, either novel images served as CS+ and familiar images as CS− or vice versa (Figure S1). Participants were instructed to make a preference judgment of each image via a two-choice button press indicating “I prefer” (press with index finger of right hand) or “I do not prefer” (press with middle finger of right hand) depending on the contingency between novelty status and reinforcement value. The contingency was randomized and indicated prior to each run by either “novelty will be rewarded if preferred” (in which case novel images served as CS+ and familiar images as CS−) or “familiarity will be rewarded if preferred” (in which case familiar images served as CS+ and novel images as CS−). Only correct “I prefer” responses following a CS+ led to a win of £0.50, whereas (incorrect) “I prefer” responses following a CS− led to a loss of £0.10. Both correct “I do not prefer” responses following a CS− and (incorrect) “I do not prefer” responses following a CS+ led to neither win nor loss. Images were presented in random order for 1 s on a gray background followed by a white fixation cross for 2 s (ISI = 3 s). Feedback about total earnings was given after each MEG scanning session (containing two blocks with each contingency), but not on a trial-by-trial basis. Prior to the experiment, the subjects were instructed to respond as accurately as possible as soon as they could confidently classify the stimuli and informed that only 20% of all earnings would be paid.

In experiment II, an MEG scanning session also contained two blocks. During each MEG scanning session, familiar and novel images were also presented randomly intermixed, and subjects were instructed before each block to indicate either (1) image novelty with their index finger and familiarity with their middle finger or (2) novelty with their middle finger and familiarity with their index finger (in both cases with the right hand). This was done to match the alternations of response contingencies across both experiments. As in experiment I, subjects were instructed to respond as accurately as possible as soon as they could confidently classify the stimuli. In both experiments, subjects were first instructed verbally, followed by written instruction. Furthermore, subjects used the same response device in both experiments to make their responses.

All images were grayscaled and normalized to a mean gray value of 127 and a standard deviation of 75 (8-bit grayscale, 0–255). None of the images depicted human beings or parts of human beings, including faces in the foreground.

### Training Sessions

In experiment I, each subject performed two training sessions (outside the MEG scanner) prior to the experiment. Similar to the actual experiment, both trainings began with a familiarization phase, during which only ten images were presented twice in a random order (duration 1.5 s; ISI = 3 s), and subjects indicated their indoor/outdoor status. Also similar to the main experiment, the familiarization was followed by a memory-based preference judgment task including familiar and novel images. Importantly, in training session 1, feedback was given on a trial-by-trial basis after each response. In training session 2, reward feedback was not given immediately after each stimulus/response. Following each training session, the subject's financial reward (maximum £1) was reported to the subject. In experiment II, subjects also received a brief training session containing ten familiar and ten novel images per response contingency block. See Supplemental Data for statistical analyses ruling out the possibility that training effects or gender contributed to the early novelty ERFs.

### MEG Methods

MEG recordings were made in a magnetically shielded room with a 275-channel Canadian Thin Films system with superconducting quantum interference device (SQUID)-based axial gradiometers (VSM MedTech Ltd.) and second-order gradients. Neuromagnetic signals were digitized continuously at a sampling rate of 600 Hz, and behavioral responses were made via an MEG-compatible response pad. Data were low-pass filtered at 120 Hz during acquisition.

### Data Analysis

Data were analyzed with SPM8 [25, 26] (Wellcome Trust Centre for Neuroimaging; see also [19, 27, 28]) and MATLAB 7 (The MathWorks).

#### Preprocessing

For each subject, data were extracted from 100 ms before to 1500 ms after stimulus onset (i.e., epoched) and baseline corrected relative to the 100 ms before stimulus onset. Epoched data were downsampled at 150 Hz and low-pass filtered at 20 Hz. Before averaging trials for each condition, simple thresholding was applied to remove artifact-containing trials with signals exceeding 2500 fT. For both experiments, only trials with correct behavioral responses were used for averaging. Epoched data were converted into Neuroimaging Informatics Technology Initiative (NIfTI) format. This produced a 3D image of channel space × time. The 2D channel space was created by projecting the sensor locations onto a plane followed by a linear interpolation to a 64 × 64 pixel grid (pixel size = 3 × 3 mm). The time dimension consisted of 241 6.67 ms samples per epoch. Finally, these images were smoothed via a Gaussian kernel (full width half maximum [FWHM]) of 6 mm and averaged across time for five time windows (85–115 ms, 115–150 ms, 150–200 ms, 200–500 ms, and 500–700 ms) prior to analysis at the second level. Thus, this produced one image per condition, time window, and subject.

#### Statistical Analysis

Each time window was analyzed via ANOVA. For experiment I, a 2 × 2 ANOVA with the factors novelty (novel, familiar) and reward (rewarding, not rewarding) was performed. For experiment II, a one-way ANOVA with the factor novelty (novel, familiar) was performed. All F-contrasts were thresholded at p = 0.005, uncorrected (F = 11.37) except for the novelty contrast in experiment I, time window 500–700 and experiment II, time window 150–200 (results for both contrasts are reported at a threshold of 0.01, uncorrected; F = 9.07). This lower threshold was motivated by a priori hypotheses regarding novelty effects in this time window. Both contrasts survived directed t-contrasts at p = 0.005 (uncorrected), which were based on our initial observation of more positive ERFs for novel items in experiment I (85–115 ms) and stronger negative ERFs for familiar items in experiment II (200–500 ms).

Finally, it should be noted that our detection of novelty effects that were earlier (85–115 ms) than in previous studies cannot be explained by methodological aspects of our analysis (e.g., a potentially higher spatiotemporal sensitivity of SPM for MEG/electroencephalography) or the specific MEG scanner, given that the same method was used in experiments I and II.

## Figures and Tables

**Table 1:** Behavioral Results

Experiment I				
		d′	β	RT (ms)
Context	novel CS+, familiar CS−	2.15 (0.55)	0.94 (0.51)	hits: 1084 (301)
				correct rejections: 1096 (286)
	familiar CS+, novel CS−	2.30 (0.64)	1.72 (2.25)	hits: 1019 (341)
				correct rejections: 1075 (357)

Experiment II				

		d′	β	RT (ms)

Context	index finger novel, middle finger familiar	2.21 (0.80)	1.45 (0.94)	hits: 1260 (316)
				correct rejections: 1226 (329)
	index finger familiar, middle finger novel	2.41 (0.96)	1.33 (1.52)	hits: 1151 (306)
				correct rejections: 1253 (359)

Accuracy did not differ between contexts and experiments as revealed by mixed-effects 2 × 2 ANOVA on d′ with the factors context (two contexts) and experiment (experiments I and II; between-subject factor). This revealed no main effect of context (F(1,26) = 2.063, p = 0.163) and no interaction between context and experiment (F(1,26) = 0.059, p = 0.809). Furthermore, a 2 × 2 ANOVA with the factor novelty (novel, familiar) and the between-subject factor experiment (experiments I and II) on reaction time (RT) showed a tendency toward an interaction between novelty and experiment (F(1,26) = 3.08, p = 0.09). Further RT analysis revealed one extreme outlier in experiment I (mean RT for novel items and mean RT for familiar items greater than three times the interquartile range; note that this subject had nonoutlying values in all other measures). Excluding this subject, the interaction between novelty and experiment approached statistical significance (F(1,25) = 4.22, p = 0.051), justifying a direct comparison between RT of both experiments. Excluding the outlier, RT for novel images in experiment I was faster than in experiment II (p = 0.011), and the same was true for familiar images (p = 0.048; both two-tailed). Response bias (β values) in both contexts and both experiments did not differ from 1. Moreover, direct comparison between β values in both contexts showed no significant difference in response bias between rewarding novel or familiar stimuli in experiment I (p > 0.2) and no effect of response finger on response bias in experiment II (p > 0.8). Mean values are shown; numbers in parentheses represent one standard deviation of the mean.

## Supplemental Data

Document S1. Supplemental Results and Discussion, Three Figures, and One Table
