# Supplementary material for: Reward Motivation Accelerates the Onset of Neural Novelty Signals in Humans to 85 Milliseconds
Source: Curr Biol. 2009 Aug 11;19(15):1294–300. doi: 10.1016/j.cub.2009.06.021 (PMC2764383; doi:10.1016/j.cub.2009.06.021)
Supplement: Document S1. Supplemental Results and Discussion, Three Figures, and One Table [file mmc1.pdf]

Current Biology, Volume *19*

Supplemental Data

Reward Motivation Accelerates the Onset of Neural Novelty Signals in Humans to 85 Milliseconds

Nico Bunzeck, Christian F. Doeller, Lluis Fuentemilla, Raymond J. Dolan, and Emrah Duzel

Supplemental Results and Discussion

We investigated to what extent the early onset novelty effect was caused by training effects across experimental runs. ERFs from temporal and frontal sensors for the earliest time-windows associated with novelty effects in both experiments (85-115ms and 200-500ms) were separated for all three runs. If training leads to an acceleration of the onset of novelty responses one would expect differences between ERFs for old and novel images as a function of runs (i.e., a run by novelty interaction). 3x2 ANOVAs with the factors run (1,2,3) and novelty (new, familiar) were performed on ERFs from (1) early time-window, temporal sensors, Experiment I, (2) early time-window, temporal sensors, Experiment II, (3) late time-window, frontal sensors, Experiment I, (4) late time-window, frontal sensors, Experiment II, (5) early time-window, frontal sensors, Experiment I, and (6) early time-window, frontal sensors, Experiment II. None of the ANOVAs revealed a statistically significant interaction between run and novelty (all $p>0.1$). Furthermore, early novelty responses over temporal sensors were not different between run 1 and run 3 in either experiment ($p>0.22$) providing further evidence that there was no effect of training on early novelty effects in either experiment. This lack of training effects also rules out the possibility that having 10 more training trials in Experiment I than in Experiment II contributed to the early onset of novelty effects in Experiment I.

Sex ratios in Experiment I (six male, eight female) and Experiment II (three male, eleven female) were different. In order to address the possibility that gender differences influenced our findings we computed the difference between ERFs for novel and familiar items extracted from temporal and frontal peaks for the early (85-115ms) and late (200-500ms) time-window and compared them between male and female participants using Mann-Whitney U-test. There was no statistically significant difference between ERFs for any of the time-windows or locations in either experiment (all $p>0.45$). Thus, our results suggest that there was no influence of gender on the early onset of novelty effects.

The topographic distribution of novelty effects were examined within experiments using 2x2 ANOVAs with the factors novelty and location (frontal vs. temporal sensors). In Experiment I for the early time-window (85-115ms) a significant interaction between novelty and location ($F(1,13)=8.16$, $p=0.013$; and a significant main effect of novelty, ($F(1,13)=6.06$, $p=0.029$) with significant novelty effects emerging only over temporal sensors ($p<0.01$) but not frontal sensors ($p>0.6$) showed that early novelty effects in Experiment I were predominant over temporal but not frontal sensors. For Experiment II this ANOVA revealed no main effects or interactions ($p>0.8$). For the later time-window (200-500ms), in Experiment II a significant interaction between location and novelty ($F(1,13)=6.51$, $p=0.024$) with significant novelty effects over frontal ($p<0.0002$) but not temporal sensors ($p>0.14$) indicated that novelty effects were expressed primarily over frontal but not temporal sensors. For Experiment I this ANOVA revealed marginally significant interactions between novelty and location ($F(1,13)=4.04$, $p=0.066$) with significant novelty responses over temporal ($p<0.05$) but not frontal sensors ($p>0.34$).

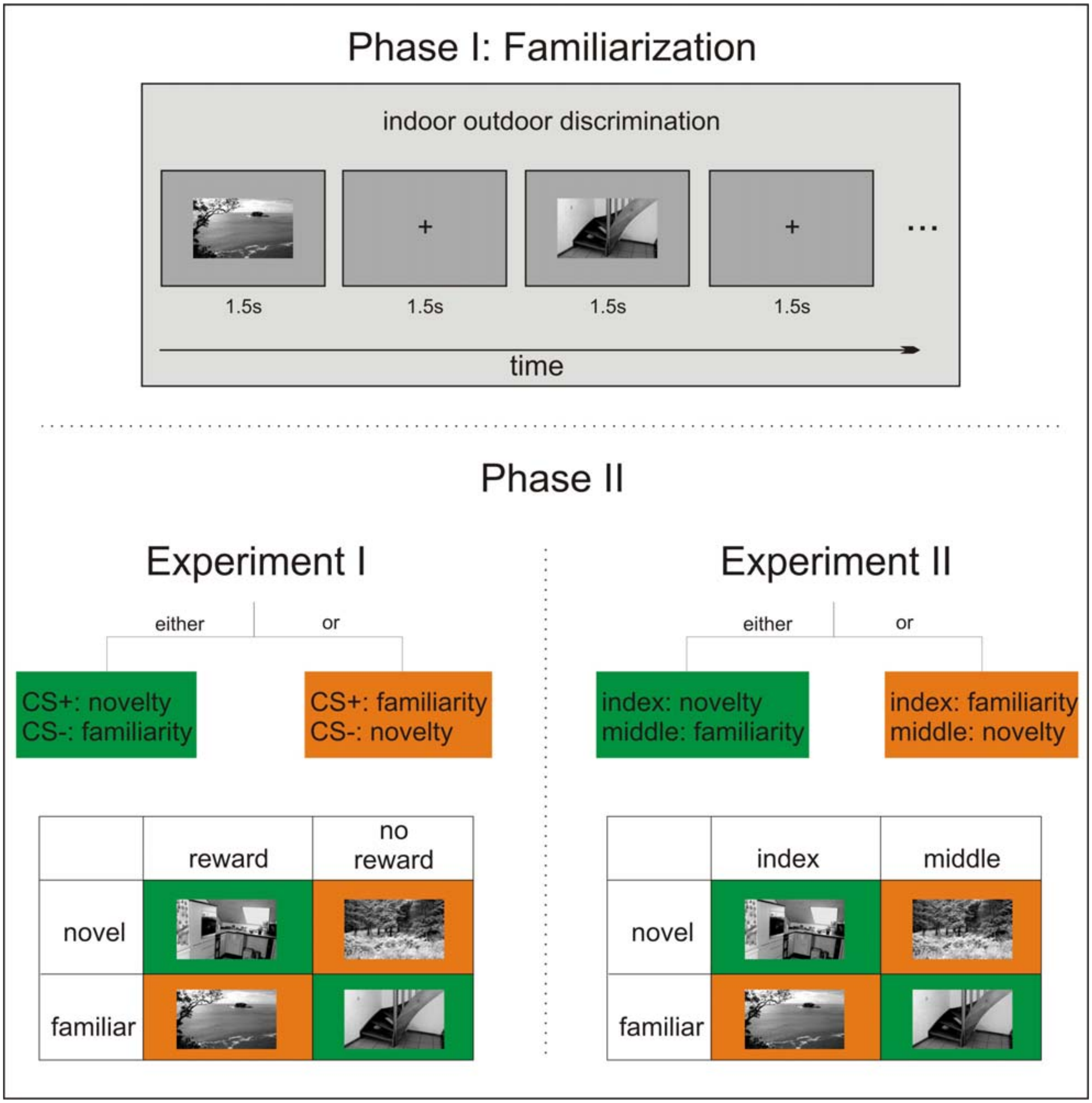

Figure S1. Experimental Design

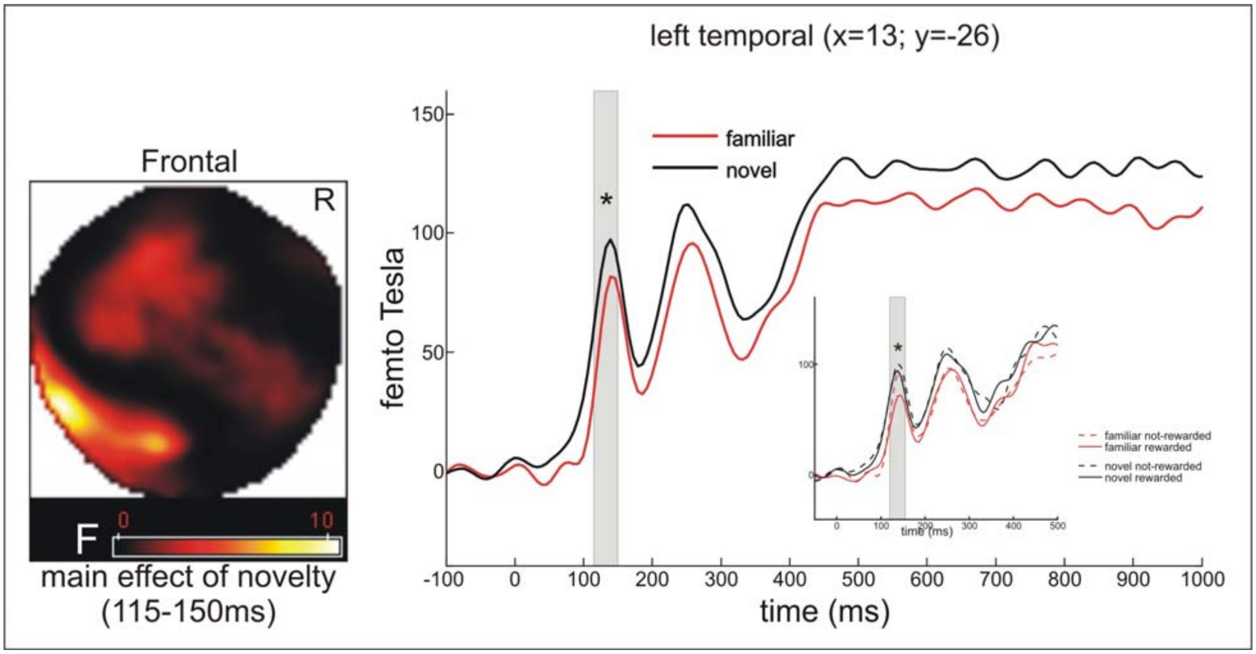

Figure S2. Experiment I Results for the Time Window 115-150 ms

ERFs distinguished between novel and familiar stimuli at 115-150ms after stimulus onset (p=0.005; uncorrected; F=11.37).

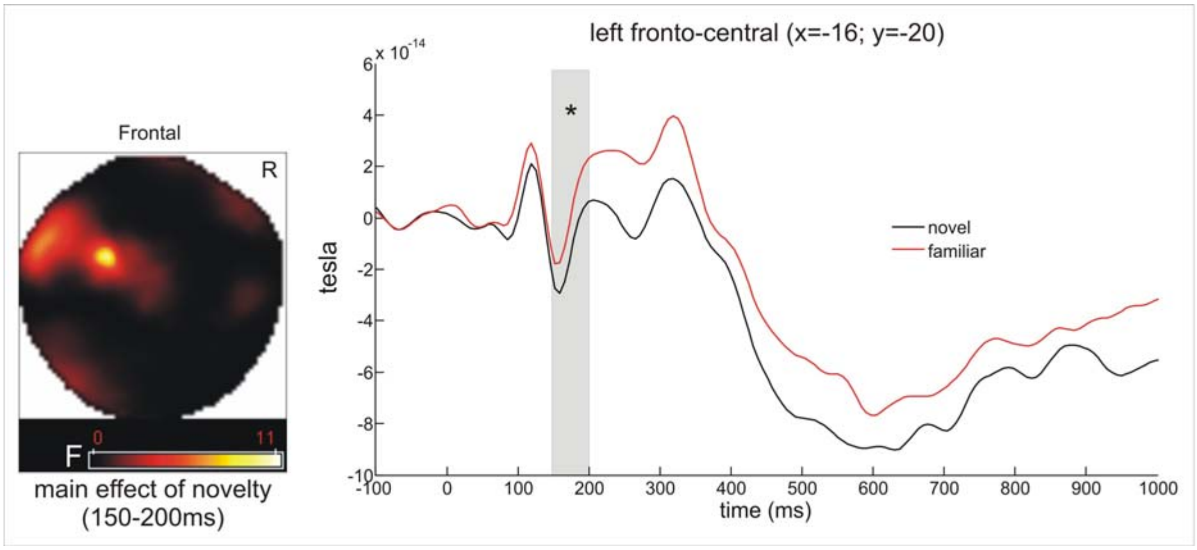

Figure S3. Experiment II Results for the Time Window 150-200 ms

ERFs distinguished between novel and familiar stimuli at 150-200ms after stimulus onset at a liberal threshold of p=0.01 (uncorrected, F=9.07).

Table S1. Experiment I and II Results

Time Window (ms)	Experiment I			Experiment II
	**Main Effect of Novelty**	**Main Effect of Reward**	**Interaction**	**Main Effect of Novelty**
85-115	x, y: 13, -19<br>(left temporal)<br>Nr. of voxel (K) = 121<br>F = 11.95<br>P_{uncorr} = 0.001	-	-	-
115-150	x, y: 13, -26<br>(left temporal)<br>Nr. of voxel (K) = 25<br>F = 10.49<br>P_{uncorr} = 0.002	-	-	-
150-200	-	-	-	*x, y: -16, 20*<br>*(left frontal)*<br>*Nr. of voxel (K) = 19*<br>*F = 11.20*<br>*P_{uncorr} = 0.005*
200-500	-	x, y: 10, -16<br>(right frontal)<br>Nr. of voxel (K) = 27<br>F = 10.42<br>P_{uncorr} = 0.003	-	x, y: -8, -11<br>(left fronto-central)<br>Nr. of voxel (K) = 32<br>F = 27.34<br>P_{uncorr} < 0.001<br><br>x, y: -12 -26<br>(left frontal)<br>Nr. of voxel (K) = 55<br>F = 15.95<br>P_{uncorr} = 0.002
500-700	*x, y: 20, -7*<br>*(left parietal)*<br>*Nr. of voxel (K) = 20*<br>*F = 8.77*<br>*P_{uncorr} = 0.005*	-	-	x, y: -7, -11<br>(left fronto-central)<br>Nr. of voxel (K) = 2<br>F = 11.86<br>P_{uncorr} = 0.004

For all time-windows of interest listed are main effects of novelty and reward and interactions for Experiment I and main effects of novelty for Experiment II. All F-contrasts were thresholded at p=0.005 (uncorrected; F=11.37) except for the two contrasts listed in italics (p=0.01, uncorrected; F=9.07). The lower threshold was motivated by a priori hypotheses and it should be noted that both contrasts survived directed t-contrast at p=0.005 (uncorrected).
